# More than Meets the Eye: Aspergillus-Related Orbital Apex Syndrome

**DOI:** 10.7759/cureus.9352

**Published:** 2020-07-23

**Authors:** Joshua A Ronen, Faizan A Malik, Catherine Wiechmann, Swapna Kolli, Raphael Nwojo

**Affiliations:** 1 Internal Medicine, Texas Tech University Health Sciences Center at Permian Basin, Odessa, USA; 2 Internal Medicine, Texas Tech University Health Sciences Center, Lubbock, USA; 3 Otolaryngology, Medical Center Health System, Odessa, USA

**Keywords:** orbital apex disorders, orbital apex syndrome, cavernous sinus syndrome, superior orbital fissure syndrome, orbital cellulitis, preseptal cellulitis, mucorales, aspergillus, jacod syndrome, phaeohyphomycosis

## Abstract

The patient is a 67-year-old Caucasian male with a past medical history of diabetes mellitus type 2, coronary artery disease (CAD) status post stent placement, renal cell carcinoma (RCC) status post left nephrectomy and bilateral adrenalectomy secondary to metastatic disease, and aspergillus pneumonia who was transferred from an outside hospital for evaluation of progressively worsening pulsating right temple and retrobulbar headache. Initial studies ruled out glaucoma, giant cell arteritis, and stroke, or aneurysmal pathology. The only positive finding was right sphenoid sinus disease on imaging that had caused bony destruction and infiltration of the right orbital apex. Broad-spectrum antibiotics were started for bacterial versus fungal sinusitis and the patient was admitted to the medical floor with consultations to Neurology, Otolaryngology (ENT), and Ophthalmology. ENT took the patient emergently to the OR. The final diagnosis was chronic aspergillus sinusitis and right-sided orbital apex syndrome (OAS). Antibiotics and antifungals were optimized by the infectious disease team. ENT also ordered steroid washouts post-operatively with budesonide and saline as well as sinus debridements every couple of weeks.

## Introduction

The orbital apex disorders include cavernous sinus syndrome (CSS), superior orbital fissure syndrome (SOFS), and orbital apex syndrome (OAS). All three disorders have varying etiologies, similar clinical manifestations, and varying degrees of severity. Thus, prompt identification is imperative for proper treatment and preservation of vision.

OAS is an extra-orbital complication of orbital (“postseptal”) cellulitis, a sight-threatening and life-threatening infection of the soft tissue posterior to the orbital septum [1]. It is much more commonly found in young children than adults. Co-existing (bacterial) pan- or ethmoid rhinosinusitis is described in 86-98% of cases [1]. Other causes of orbital cellulitis include ophthalmic surgery, peribulbar anesthesia, orbital trauma with a fracture or foreign body, and dental or middle ear infections. Blindness can occur by way of extra-orbital extension of the infection to the orbital apex; this is known as orbital apex syndrome (OAS) or Jacod syndrome. While the causative organisms are often hard to identify, *Staphylococcus aureus *(*S. aureus*), *Streptococcus anginosus*, non-typeable *Haemophilus influenzae, Mucorales*, and* Aspergillus *spp. have been identified in association with orbital cellulitis [1]. The latter two microbes are more often found in association with OAS in immunocompromised patients [2]. Less than 5% of blood cultures come back positive in adults and wound cultures have been noted to come back as polymicrobial only in pediatric cases. 

Preseptal cellulitis (also known as periorbital cellulitis) which involves the soft tissues anterior to the orbital septum (i.e. including the eyelid), is much more common than orbital cellulitis [1]. While preseptal cellulitis is not known to be sight or life-threatening, sometimes the diagnosis can remain unclear and patients should be treated as if they have orbital cellulitis for this reason until it can be definitively ruled out.

## Case presentation

The patient is a 67-year-old white male with a past medical history of diabetes mellitus type 2 (HbA1c of 8.8%), hypertension, hypothyroidism, coronary artery disease (CAD) status post stent placement, renal cell carcinoma status post left nephrectomy and bilateral adrenalectomy secondary to metastatic disease, history of aspergillus pneumonia, left occipital meningioma, and benign prostatic hyperplasia who was admitted to the medical floor for further workup and management of a severe right temple and retrobulbar headache. Two weeks prior to admission he endorsed having a sinus infection from which he still had persistent pain and congestion. These symptoms were also accompanied by intermittent episodes of diplopia, photophobia, and tearing of the right eye for three weeks prior to admission. Examination of the affected eye revealed sinus tenderness, chemosis, periorbital tenderness and proptosis, and lateral gaze palsy. Extraocular movements of the left eye were intact. Pupils were also equal and reactive to light and accommodation bilaterally.

A CT scan of the head was obtained and came negative for any acute process. The patient tested negative for giant cell arteritis and glaucoma. CT scan of the orbits without contrast showed right sphenoid sinus disease that had caused bony destruction and likely infectious infiltration of the right orbital apex. Ophthalmology, Infectious Disease, Neurology, and ENT consults were obtained. His initial antibiotic regimen consisted of intravenous (IV) vancomycin and piperacillin-tazobactam. Piperacillin-tazobactam was changed to meropenem and amphotericin by the Infectious Disease team as there was suspicion for bacterial versus fungal sinusitis (especially rhinocerebral mucormycosis given his uncontrolled diabetes). Per ENT, biopsy results of his sinuses status post initial sinus debridement revealed fungal debris which was confirmed to be *Aspergillus *spp. In light of these findings, IV amphotericin was transitioned to isavuconazole. MRI scans of the brain and neck including angiography and venography were negative for any aneurysmal pathology and venous sinus thrombosis. Chronic paranasal sinusitis was the only positive finding. A lumbar puncture was negative. During his hospital course, an edema around the patient's right eye subsided although the lateral gaze palsy remained. Ophthalmology did not appreciate any papilledema on fundoscopic examination. They did not recommend any acute intervention beyond outpatient follow-up after discharge from the hospital. ENT also ordered steroid washouts post-operatively with budesonide and saline as well as sinus debridements every couple of weeks. His final antibiotic regimen per the Infectious Disease team consisted of IV vancomycin and cefepime for six weeks as well as per oral (PO) voriconazole for six months.

## Discussion

**Figure 1:**
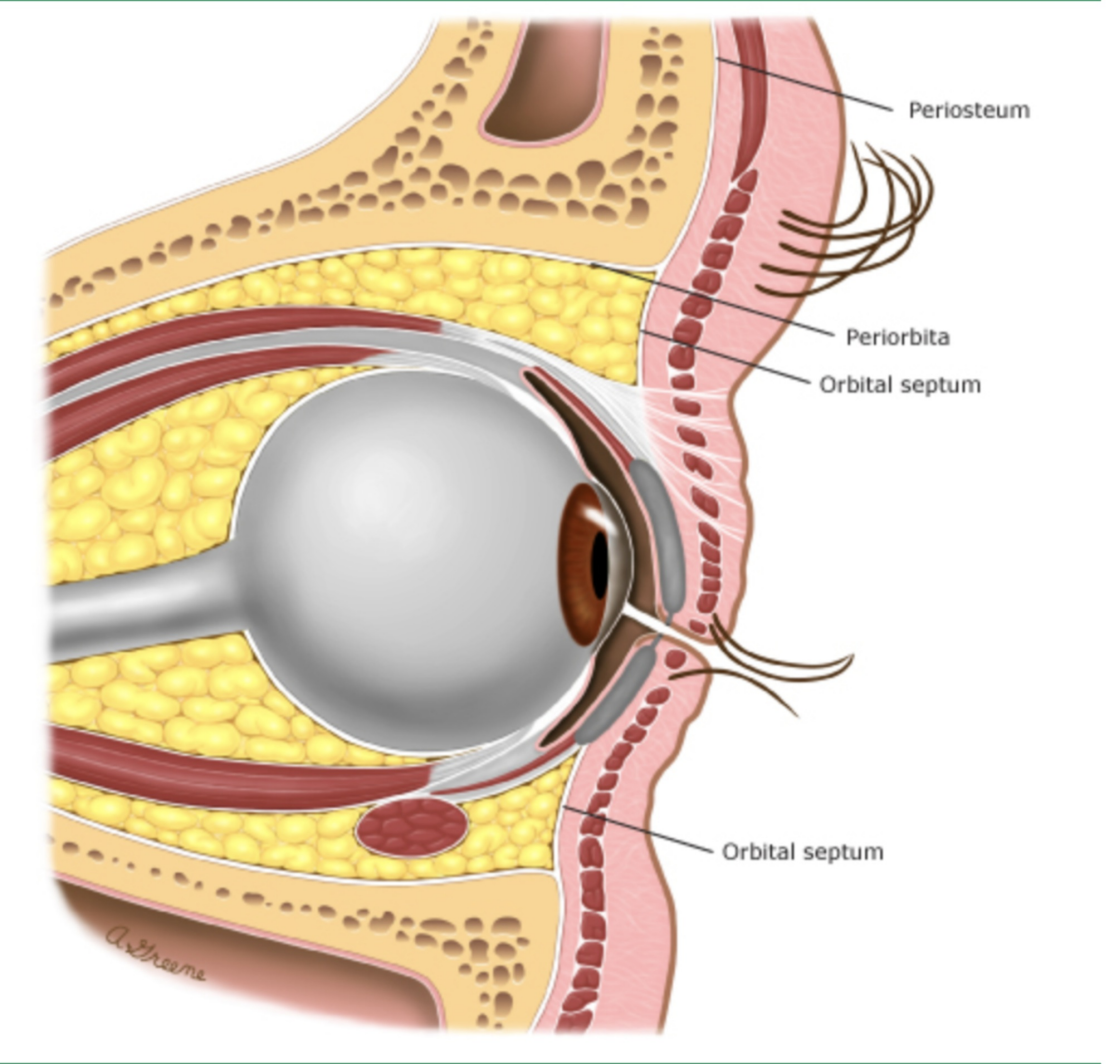
The orbital septum

**Figure 2:**
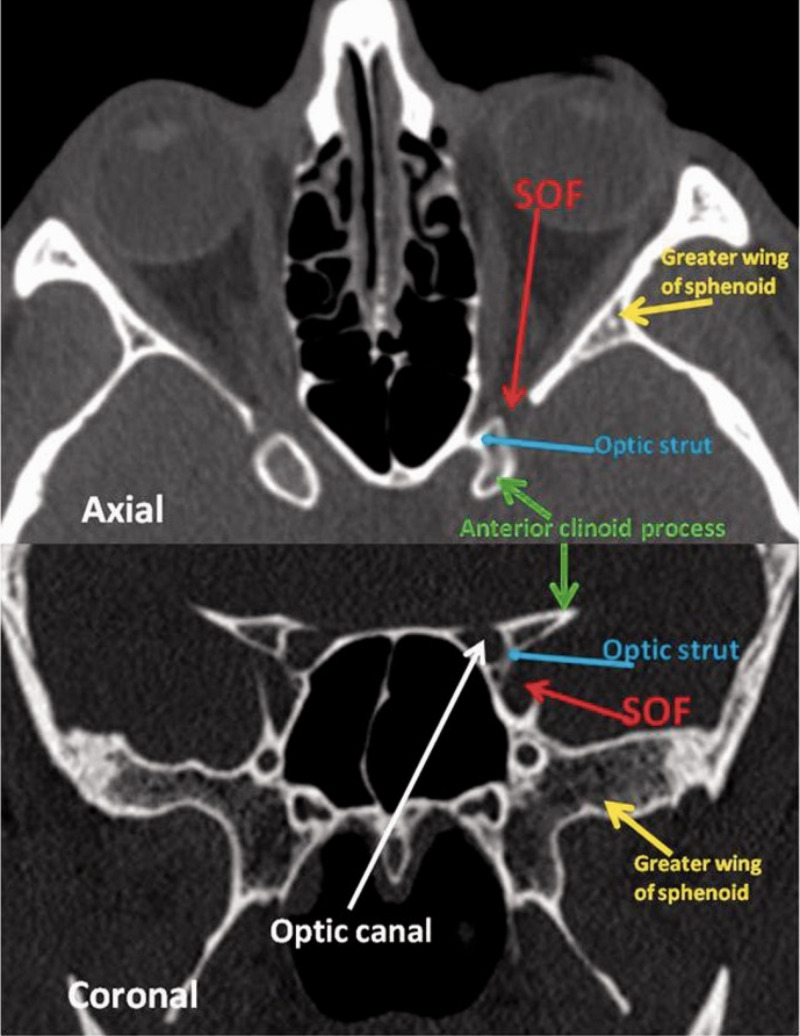
Orbital apex anatomy The axial and coronal CT slices demonstrate the useful clinical anatomy of the orbital apex. Red arrow: SOF; yellow arrow: Greater wing of sphenoid; blue arrow: optic strut; green arrow: anterior clinoid process; white arrow: optic canal; SOF: superior orbital fissure. This image was obtained with consent from The Neuroradiology Journal [3].

The orbit itself is a cone-shaped structure with its apex within the skull (Figures 1-2). The orbital apex disorders as Cox et al. describe include OAS, CSS, and SOFS [2]. They can be progressive in nature with SOFS developing into OAS or CSS. OAS, CSS, and SOFS share similar etiologies, symptomatologies, diagnostic evaluations, and management strategies. CSS results from compression of the sinuses themselves, and SOFS results from a lesion immediately anterior to the orbital apex (Figure 2). Etiologies of each of these orbital apex disorders could be neoplastic, inflammatory, developmental, traumatic, or infectious as in this case. OAS is an infectious complication of orbital cellulitis, involving soft tissues posterior to the orbital septum (Figure 1). Orbital cellulitis is typically precipitated by ethmoid sinusitis. Notwithstanding, both preseptal cellulitis and orbital cellulitis have different clinical implications and it is important to distinguish between them [2]. Orbital cellulitis presents the greater emergency, with an immediate threat to vision as well as to life. Preseptal cellulitis mainly presents with eyelid swelling with or without erythema and may be associated with fever and leukocytosis. Clinical features of orbital cellulitis include these as per Gappy et al. along with eye pain and tenderness, pain with extraocular eye movements and proptosis [1]. Ophthalmoplegia with or without diplopia, vision impairment (manifested by an afferent pupillary defect), and chemosis may also be present. Diagnosis of orbital cellulitis is made via contrast-enhanced CT scan of the orbits and sinuses. Complications of orbital cellulitis include subperiosteal cellulitis, orbital abscess, visual loss, and intracranial extension [1]. The main objective with orbital cellulitis is the preservation of vision. Extraorbital extension of orbital cellulitis presents as OAS. Intracranial extension of orbital cellulitis can cause subdural empyemas, epidural abscesses, meningitis, and CSS. 

*Mucor*, *Aspergillus spp*., and *Mycobacterium tuberculosis* have been identified as the main causative pathogens of orbital cellulitis which can involve extra-orbital extension resulting in OAS [1-2]. While such cases are rare, Goyal et al. explain that patients are suspected to be already predisposed to immunodeficiency via chronic diseases including but not limited to the pancreas (diabetes), kidneys (renal acidoses), and human immunodeficiency virus (HIV) [2]. Many early infections are confined to the maxillary and sphenoid sinus [1]. In rare cases, the infection invades through the sphenoid bone, which results in OAS [3]. OAS itself is known to cause blindness, loculations of infection within the intracranial compartment, and cavernous or dural venous sinus thromboses. Startlingly enough, there are no other alarming signs of inflammation. Emergent surgical intervention paired with long-term intravenous antibiotics is warranted to preserve vision and avoid deleterious insult to the orbital compartment [3-4].

Management

If it is unclear whether a patient has preseptal cellulitis or orbital cellulitis, their response to antibiotic therapy can also help confirm the diagnosis beyond the history of present illness, physical examination, and diagnostic studies. Parenteral broad-spectrum antibiotic therapy against *S. aureus* (including methicillin-resistant *Staphylococcus aureus*), *Streptococci*, and gram-negative bacilli such as Pseudomonas aeruginosa should be started. If there is lack of improvement in signs and symptoms 24-48 hours after the initiation of such therapy in addition to worsening visual acuity or pupillary changes, absolute neutrophil count (ANC) > 10,000 cells/uL, evidence of abscess greater than 1 cm in diameter, and limited extra-ocular muscle movements, orbital cellulitis should be suspected. Management of this should include consultation of an ophthalmologist and an ENT. Repeat imaging and endoscopic nasal surgery to biopsy should follow closely after [1, 4-5]. For uncomplicated infections, Gappy et al. recommend that antibiotics should be continued until there is complete resolution: this can range up to at least two to three weeks of IV and PO antibiotic therapy [1]. For complicated infections such as severe ethmoid sinusitis accompanied by bony destruction of the sinus, at least four weeks of antibiotic coverage is recommended [1-2]. The transition from IV to PO therapy is at the discretion of Infectious Disease specialists, who also need to decide whether or not certain patients are candidates for peripherally inserted central catheters (PICCs) or midline catheters to complete IV antibiotic infusions as an outpatient. 

**Figure 3:**
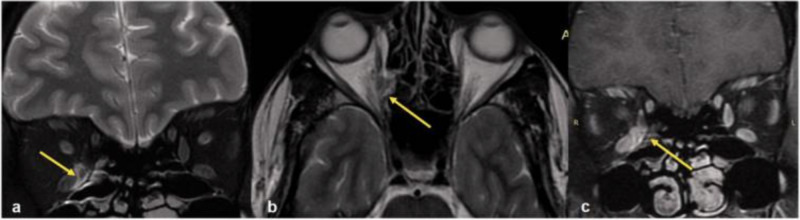
MRI of orbit Coronal (a), axial (b) T2W MR image shows a hyperintense lesion medial to the medial rectus muscle (yellow arrow) extending to the medial orbital wall forming sub-periosteal abscess and extending laterally into the intraconal fat. Post-contrast coronal T1W image (c) shows the enhancement (yellow arrow). This was a case of orbital cellulitis. T2W: T2-weighted; T1W: T1-weighted This image was obtained with consent from The Neuroradiology Journal [3].

Fungal rhinosinusitis is more common in immunocompromised patients. Inhaled fungal spores can commonly colonize the sinuses and lungs, but that does not mean that they will cause overt disease. In these patients with immunocompromising conditions such as diabetes or a history of cancer such as in the patient described, a more aggressive and invasive disease course is likely [2, 6]. Acute infections are caused by *Aspergillus, Fusarium*, and *Mucorales* while chronic indolent infections are caused by dematiaceous (brown-black molds also known as phaeohyphomycosis) such as *Bipolaris*, *Curvularia*, and *Alternaria spp*. Cox et al. point out that the latter variant can also be caused by *Aspergillus* and *Scedosporium apiospermum *complex [2]. With suggestive symptoms in immunocompromised patients such as fever, facial pain, nasal congestion, vision impairment, and altered sensorium, CT imaging should be obtained immediately. If any abnormalities are detected, MRIs should be performed (Figures 2-3). Nasal endoscopy and possible radical surgical debridement by ENTs are needed to assess signs of necrosis otherwise indicative of rhinocerebral mucormycosis, a deadly infection. In the presence of a perforated nasal septum or palatal or gingival eschars (ie. necrosis), fungi are understood to have already invaded the intravascular space of the maxillofacial region [2]. The diagnosis of invasive fungal rhinosinusitis is confirmed only on histopathology. Empiric IV antifungal therapy typically includes lipid formulations of amphotericin B 5 mg/kg daily or voriconazole 6 mg/kg every 12 hours for two doses followed by 4 mg/kg every 12 hours after that, if mucormycosis* *is effectively ruled out (*Mucorales *is generally known to be resistant to triazole antifungal agents such as voriconazole) [2]. Isavuconazole 200 mg every eight hours for two days (PO or IV) followed by once daily thereafter can be used in the setting of intolerance to voriconazole. Nonetheless, the patient's own clinical profile and the agent's own side-effect profiles should be considered in the decision-making process to optimize the chosen antifungal regimen. The response to therapy should also be monitored closely as that will ultimately determine when it is safe to transition from IV to PO therapy. PO dosing for voriconazole is 200 mg twice daily. Acute, invasive, and indolent rhinosinusitis secondary to *Aspergillus* is responsive to voriconazole. All in all, suppressive therapy with amphotericin, voriconazole, or the echinocandins such as micafungin or caspofungin (if the disease is severe enough) can last up to six months and immunocompromised patients may require longer. As far as antifungal choice is concerned, the financial burden on the patient, if long-term outpatient antibiotic therapy is required, also needs to be considered.

## Conclusions

Prompt identification is imperative for proper treatment and preservation of vision in the setting of orbital apex ("Jacod") syndrome. With most patients displaying concurrent symptoms of sinus disease and vision impairment, a work-up should be initiated including blood tests, cultures, and radiography. Even in the setting of preseptal cellulitis, broad-spectrum IV antibiotic therapy should be started rapidly and response to therapy should be monitored closely. Lack of response or worsening signs and symptoms of ongoing disease should necessitate involvement of Infectious Disease, ENT, and Ophthalmology specialists. Special consideration should be given to patients that are immunocompromised (such as those who are diabetic or with a history of cancer or immunodeficiency) as they are at higher risk of invasive disease with more virulent organisms and fungi such as *Aspergillus spp*. The primary objective is ruling out necrotizing infection such as rhinocerebral mucormycosis with emergent nasal endoscopy if such is suspected. Fungal rhinosinusitis infection can be acute and invasive or chronic and indolent, but equally dangerous. In consideration of this, sinus biopsies and serial debridements can facilitate the overall management of the underlying illness. With cases of orbital cellulitis complicated by extra-orbital extension such as in the patient described, Infectious Disease specialists and Care Coordination teams (ie. social workers) in hospital should work together to prepare and manage long-term antibiotic coverage, arrange follow-ups and central vascular access, and ensure the primary medical teams remain appraised of the patient's disease course after discharge from the acute care setting.
